# Supplementary material for: Enhancing transparency in reporting the synthesis of qualitative research: ENTREQ
Source: BMC Med Res Methodol. 2012 Nov 27;12:181. doi: 10.1186/1471-2288-12-181 (PMC3552766; doi:10.1186/1471-2288-12-181)
Supplement: Additional file 1 — Search strategy. [file 1471-2288-12-181-S1.doc]

**W1. Search strategy**

(synthesis and qualitative studies).tw

(synthesis and qualitative research).tw

(systematic review and qualitative studies).tw

(systematic review and qualitative research).tw

critical interpretive synthesis.tw

(meta and ethnography).tw

meta-study.tw

thematic synthesis.tw

meta-narrative.tw

textual narrative synthesis.tw

framework synthesis.tw

(grounded theory and synthesis).tw.

(triangulation and qualitative and synthesis).tw
