# Supplementary material for: Enhancing transparency in reporting the synthesis of qualitative research: ENTREQ
Source: BMC Med Res Methodol. 2012 Nov 27;12:181. doi: 10.1186/1471-2288-12-181 (PMC3552766; doi:10.1186/1471-2288-12-181)
Supplement: Additional file 2 — Search results. [file 1471-2288-12-181-S2.doc]

**W2. Search results**
