# Supplementary material for: Enhancing transparency in reporting the synthesis of qualitative research: ENTREQ
Source: BMC Med Res Methodol. 2012 Nov 27;12:181. doi: 10.1186/1471-2288-12-181 (PMC3552766; doi:10.1186/1471-2288-12-181)
Supplement: Additional file 3 — Pilot test: assessment of 40 published synthesis of qualitative research using the ENTREQ Statement. [file 1471-2288-12-181-S3.doc]

**W3. Pilot test: assessment of 4**0 published synthesis of qualitative research using the ENTREQ Statement

|  | **References** | | | | | | | | | | | | | | | | | | | | | | | | | | | | | | | | | | | | | | | |
| --- | --- | --- | --- | --- | --- | --- | --- | --- | --- | --- | --- | --- | --- | --- | --- | --- | --- | --- | --- | --- | --- | --- | --- | --- | --- | --- | --- | --- | --- | --- | --- | --- | --- | --- | --- | --- | --- | --- | --- | --- |
| **Item** | [^1^](#_ENREF_1) | [^2^](#_ENREF_2) | [^3^](#_ENREF_3) | [^4^](#_ENREF_4) | [^5^](#_ENREF_5) | [^6^](#_ENREF_6) | [^7^](#_ENREF_7) | [^8^](#_ENREF_8) | [^9^](#_ENREF_9) | [^10^](#_ENREF_10) | [^11^](#_ENREF_11) | [^12^](#_ENREF_12) | [^13^](#_ENREF_13) | [^14^](#_ENREF_14) | [^15^](#_ENREF_15) | [^16^](#_ENREF_16) | [^17^](#_ENREF_17) | [^18^](#_ENREF_18) | [^19^](#_ENREF_19) | [^20^](#_ENREF_20) | [^21^](#_ENREF_21) | [^22^](#_ENREF_22) | [^23^](#_ENREF_23) | [^24^](#_ENREF_24) | [^25^](#_ENREF_25) | [^26^](#_ENREF_26) | [^27^](#_ENREF_27) | [^28^](#_ENREF_28) | [^29^](#_ENREF_29) | [^30^](#_ENREF_30) | [^31^](#_ENREF_31) | [^32^](#_ENREF_32) | [^33^](#_ENREF_33) | [^34^](#_ENREF_34) | [^35^](#_ENREF_35) | [^36^](#_ENREF_36) | [^37^](#_ENREF_37) | [^38^](#_ENREF_38) | [^39^](#_ENREF_39) | [^40^](#_ENREF_40) |
| **1** | ● | ● | ● | ● | ● | ● | ● | ● | ● | ● | ● | ● | ● | ● | ● | ● | ● | ● | ● | ● | ● | ● | ● | ● | ● | ● | - | ● | ● | ● | ● | ● | ● | ● | ● | ● | ● | ● | ● | ● |
| **2** | ● | ● | ● | ● | ● | ● | ● | ● | ● | ● | ● | ● | - | ● | ● | ● | ● | ● | ● | ● | ● | ● | ● | ● | ● | ● | ● | ● | ● | - | ● | ● | ● | ● | ● | ● | ● | ● | ● | ● |
| **3** | ● | ● | ● | ● | ● | ● | ● | ● | ● | ● | ● | ● | ● | ● | ● | ● | ● | ● | ● | ● | ● | ● | ● | ● | ● | ● | ● | - | ● | ● | ● | ● | ● | ● | ● | ● | ● | ● | ● | ● |
| **4** | ● | ● | ● | ● | ● | ● | ● | ● | ● | ● | ● | - | ● | ● | ● | ● | ● | ● | ● | ● | ● | ● | ● | ● | ● | ● | ● | - | ● | ● | ● | - | ● | ● | ● | ● | ● | ● | - | ● |
| **5** | ● | ● | ● | ● | ● | ● | ● | ● | ● | ● | ● | - | ● | ● | ● | ● | ● | ● | ● | ● | ● | ● | ● | ● | ● | ● | - | - | ● | ● | ● | ● | ● | ● | - | ● | ● | ● | - | ● |
| **6** | - | - | ● | - | ● | ● | ● | ● | - | ● | ● | - | ● | ● | ● | ● | ● | ● | ● | ● | ● | ● | ● | ● | ● | ● | - | - | ● | ● | ● | - | ● | - | ● | - | ● | - | - | ● |
| **7** | ● | - | - | - | ● | ● | - | ● | ● | ● | ● | - | ● | - | ● | ● | - | - | - | - | ● | ● | ● | - | - | - | - | - | ● | ● | ● | - | ● | - | ● | - | ● | ● | - | ● |
| **8** | ● | ● | ● | ● | ● | ● | ● | ● | ● | ● | ● | ● | ● | - | ● | ● | ● | - | - | ● | ● | ● | ● | ● | ● | ● | - | ● | ● | ● | - | - | ● | - | ● | ● | ● | ● | ● | ● |
| **9** | - | - | ● | - | ● | ● | - | ● | ● | ● | ● | - | - | - | ● | ● | ● | ● | ● | - | ● | ● | ● | ● | ● | ● | ● | ● | ● | ● | - | - | ● | ● | ● | - | ● | ● | - | ● |
| **10** | ● | - | ● | - | ● | - | ● | ● | ● | ● | - | - | ● | - | - | ● | ● | - | - | ● | ● | ● | ● | ● | ● | ● | - | ● | - | ● | ● | ● | ● | ● | ● | ● | ● | ● | - | ● |
| **11** | ● | - | ● | - | ● | ● | - | ● | ● | ● | ● | - | ● | ● | - | ● | ● | - | - | ● | ● | ● | ● | ● | ● | ● | ● | - | ● | - | ● | ● | ● | ● | ● | - | ● | ● | - | ● |
| **12** | - | - | - | - | - | - | - | ● | ● | ● | ● | - | ● | - | - | ● | ● | - | - | ● | ● | ● | - | ● | - | ● | ● | - | ● | - | ● | - | ● | ● | ● | - | ● | ● | - | ● |
| **13** | ● | ● | ● | - | ● | - | ● | ● | ● | ● | ● | - | ● | - | - | ● | - | - | - | ● | ● | ● | - | ● | - | ● | - | - | ● | - | - | - | ● | - | - | ● | ● | ● | - | - |
| **14** | - | ● | - | ● | ● | ● | ● | ● | ● | ● | ● | ● | ● | ● | - | ● | ● | - | - | - | ● | ● | ● | ● | ● | ● | - | - | ● | ● | ● | - | ● | ● | - | - | ● | ● | - | ● |
| **15** | - | - | - | - | - | ● | - | ● | ● | ● | - | - | - | - | - | ● | - | - | - | ● | - | ● | - | ● | ● | - | - | - | - | - | - | ● | ● | ● | - | - | ● | ● | - | - |
| **16** | - | - | - | - | - | ● | ● | ● | - | ● | ● | - | ● | - | - | - | - | - | - | ● | ● | - | - | - | - | - | ● | ● | ● | - | ● | - | - | ● | ● | ● | ● | ● | ● | - |
| **17** | - | - | ● | ● | ● | ● | ● | ● | ● | ● | ● | - | - | ● | ● | - | ● | ● | - | ● | ● | ● | ● | ● | - | ● | ● | - | ● | - | - | ● | ● | ● | ● | - | ● | ● | ● | ● |
| **18** | ● | ● | ● | ● | ● | ● | ● | ● | ● | ● | ● | - | - | ● | ● | ● | ● | ● | ● | ● | ● | ● | ● | - | - | ● | ● | ● | ● | - | ● | ● | ● | ● | ● | ● | ● | ● | ● | ● |
| **19** | ● | ● | ● | ● | ● | ● | ● | ● | ● | ● | ● | - | ● | ● | ● | ● | ● | ● | ● | ● | ● | ● | ● | ● | ● | - | - | ● | - | ● | ● | ● | ● | ● | ● | ● | ● | ● | ● | ● |
| **20** | ● | ● | - | ● | ● | - | ● | ● | ● | ● | - | ● | - | ● | ● | - | - | ● | - | ● | ● | - | - | ● | ● | ● | ● | - | - | ● | ● | ● | ● | - | ● | ● | ● | ● | ● | - |
| **21** | ● | ● | ● | ● | ● | ● | ● | ● | ● | ● | ● | ● | ● | ● | ● | ● | ● | ● | ● | ● | ● | ● | ● | ● | ● | ● | ● | ● | ● | ● | ● | ● | ● | ● | ● | ● | ● | ● | ● | ● |

(●), ENTREQ item was reported by the corresponding article; (-) not stated or not applicable

**References**

1. Howard AF, Balneaves LG, Bottorff JL. Ethnocultural women's experiences of breast cancer: a qualitative meta-study. Cancer Nursing 2007;30(4):E27-35.

2. Schlomann P, Schmitke J. Lay beliefs about hypertension: an interpretive synthesis of the qualitative research. Journal of the American Academy of Nurse Practitioners 2007;19(7):358-67.

3. Steeman E, de Casterle BD, Godderis J, Grypdonck M. Living with early-stage dementia: a review of qualitative studies. Journal of Advanced Nursing 2006;54(6):722-38.

4. Kearney MH. Enduring love: a grounded formal theory of women's experience of domestic violence. Res Nurs Health 2001;24:270-82.

5. McInnes E, Seers K, Tutton L. Older people's views in relation to risk of falling and need for intervention: a meta-ethnography. Journal of Advanced Nursing 2011.

6. Ridd M, Shaw A, Lewis G, Salisbury C. The patient-doctor relationship: a synthesis of the qualitative literature on patients' perspectives. British Journal of General Practice 2009;59(561):e116-33.

7. Lipworth WL, Davey HM, Carter SM, Hooker C, Hu W. Beliefs and beyond: what can we learn from qualitative studies of lay people's understandings of cancer risk? Health Expectations 2010;13(2):113-24.

8. Ring N, Jepson R, Hoskins G, Wilson C, Pinnock H, Sheikh A, et al. Understanding what helps or hinders asthma action plan use: A systematic review and synthesis of the qualitative literature Patient Education & Counseling 2011.

9. Tierney S, Mamas M, Skelton D, Woods S, Rutter MK, Gibson M, et al. What can we learn from patients with heart failure about exercise adherence? A systematic review of qualitative papers. Health Psychol 2011.

10. Rees R, Oliver K, Woodman J, Thomas J. The views of young children in the UK about obesity, body size, shape and weight: a systematic review. BMC Public Health 2011.

11. Feder G, Hutson M, Ramsay J, Taket AR. Women exposed to intimate partner violence: expectations and experiences when they encounter health care professionals: a meta-analysis of qualitative studies. Archives of Internal Medicine 2006;166(1):22-37.

12. Berry C, Hayward M. What Can Qualitative Research Tell Us about Service User Perspectives of CBT for Psychosis? A Synthesis of Current Evidence. Behav Cogn Psychother. 2011.

13. Giacomini M, Cook D, DeJean D. Life support decision making in critical care: Identifying and appraising the qualitative research evidence. Critical Care Medicine 2009;37(4):1475-82.

14. Khan N, Bower P, Rogers A. Guided self-help in primary care mental health: meta-synthesis of qualitative studies of patient experience. British Journal of Psychiatry 2007;191:206-11.

15. Edwards M, Davies M, Edwards A. What are the external influences on information exchange and shared decision-making in healthcare consultations: a meta-synthesis of the literature. Patient Education & Counseling 2009;75(1):37-52.

16. Flemming K. The use of morphine to treat cancer-related pain: a synthesis of quantitative and qualitative research. Journal of Pain & Symptom Management 2010;39(1):139-54.

17. Fisher HR, McKevitt C, Boaz A. Why do parents enrol their children in research: a narrative synthesis? J Med Ethics 2011.

18. Finfgeld-Connett D. Model of therapeutic and non-therapeutic responses to patient aggression. Issues in Mental Health Nursing 2009;30(9):530-7.

19. Thorne S, Paterson B, Acorn S, Canam C, Joachim G, Jillings C. Chronic illness experience: insights from a metastudy. Qual Health Res 2002;12(4):437-52.

20. Harden A, Brunton G, Fletcher A, Oakley A. Teenage pregnancy and social disadvantage: systematic review integrating controlled trials and qualitative studies. BMJ 2009;339:b4254.

21. Mills E, Jadad AR, Ross C, Wilson K. Systematic review of qualitative studies exploring parental beliefs and attitudes toward childhood vaccination identifies common barriers to vaccination. Journal of Clinical Epidemiology 2005;58(11):1081-8.

22. Walter FM, Emery J, Braithwaite D, Marteau TM. Lay understanding of familial risk of common chronic diseases: a systematic review and synthesis of qualitative research. Annals of Family Medicine 2004;2(6):583-94.

23. Pound P, Britten N, Morgan M, Yardley L, Pope C, Daker-White G, et al. Resisting medicines: a synthesis of qualitative studies of medicine taking. Social Science & Medicine 2005;61(1):133-55.

24. Morton RL, Tong A, Howard K, Snelling P, Webster AC. The views of patients and carers in treatment decision making for chronic kidney disease: systematic review and thematic synthesis of qualitative studies. BMJ 2010;340:c112.

25. Cullen DL, Stiffler D. Long-term oxygen therapy: review from the patients' perspective. Chronic Respiratory Disease 2009;6(3):141-7.

26. Downe S, Finlayson K, Walsh D, Lavender T. 'Weighing up and balancing out': a meta-synthesis of barriers to antenatal care for marginalised women in high-income countries. BJOG: An International Journal of Obstetrics & Gynaecology 2009;116(4):518-29.

27. Barroso J, Powell-Cope GM. Metasynthesis of qualitative research on living with HIV infection. Qual Health Res 2000;10(3

):340-53.

28. Jensen LA, Allen MN. A synthesis of qualitative research on wellness-illness. Qualitative Health Research 1994;4(4):349-69.

29. Sim J, Madden S. Illness experience in fibromyalgia syndrome: a metasynthesis of qualitative studies. Soc Sci Med 2008;67:57-67.

30. Evans D, Fitzgerald M. The experience of physical restraint: a systematic review of qualitative research. Contemporary Nurse 2002;13(2-3):126-35.

31. Campbell R, Pound P, Pope C, Britten N, Pill R, Morgan M, et al. Evaluating meta-ethnography: a synthesis of qualitative research on lay experiences of diabetes and diabetes care. Social Science & Medicine 2003;56(4):671-84.

32. Thomas J, Harden A. Methods for the thematic synthesis of qualitative research in systematic reviews. BMC Medical Research Methodology 2008;8:45.

33. Tong A, Morton R, Howard K, Craig JC. Adolescent experiences following organ transplantation: a systematic review of qualitative studies. Journal of Pediatrics 2009;155(4):542-9.

34. Dixon-Woods M, Cavers D, Agarwal S, Annandale E, Arthur A, Harvey J, et al. Conducting a critical interpretive synthesis of the literature on access to healthcare by vulnerable groups. BMC Medical Research Methodology 2006;6:35.

35. Munro SA, Lewin SA, Smith HJ, Engel ME, Fretheim A, Volmink J. Patient adherence to tuberculosis treatment: a systematic review of qualitative research. PLoS Medicine / Public Library of Science 2007;4(7):e238.

36. Smith LK, Pope C, Botha JL. Patients' help-seeking experiences and delay in cancer presentation: a qualitative synthesis. Lancet 2005;366(9488):825-31.

37. Tong A, Howell M, Wong G, Webster AC, Howard K, Craig JC. The perspectives of kidney transplant recipients on medicine taking: a systematic review of qualitative studies. Nephrology Dialysis Transplantation 2011;26(1):344-54.

38. Smithson J, Britten N, Paterson C, Lewith G, Evans M. The experience of using complementary therapies after a diagnosis of cancer: A qualitative synthesis. Health 2010.

39. Beck CT. A metaethnography of traumatic childbirth and its aftermath: amplifying causal looping. Qual Health Res 2011;21(3):301-11.

40. Lakeman R, FitzGerald M. How people live with or get over being suicidal: a review of qualitative studies. Journal of Advanced Nursing 2008;64(2):114-26.
